# The mitochondrial genome of *Milvus migrans* (Aves, Accipitriformes, Accipitridae), an endangered species from South Korea

**DOI:** 10.1080/23802359.2018.1450678

**Published:** 2018-04-23

**Authors:** Hey Sook Jeon, Hyeona Myeong, Seung-Gu Kang, Jung A Kim, Sang-Hwa Lee, Mu-Yeong Lee, Junghwa An

**Affiliations:** a Animal Resources Division, National Institute of Biological Resources, Incheon, Republic of Korea;; b Department of Plant & Environmental Science, College of Agriculture & Life Science Hankyong National University, Anseong, Republic of Korea;; c Graduate Program in Cellular Biology and Genetics, College of Medicine, Chungbuk National University, Cheongju, Republic of Korea;; d DNA Analysis Division, Seoul Institute, National Forensic Service, Seoul, Republic of Korea

**Keywords:** *Milvus migrans*, mitochondrial genome, black kite, next generation sequencing

## Abstract

The complete mitochondrial genome sequence of black kite, *Milvus migrans*, one of the most common diurnal raptor, was characterized using next generation sequencing. The whole genome size was 18,016 bp and consisted of 13 protein-coding genes, 22 tRNAs, 2 rRNAs, a putative control region (CR), and a second control region (pseudo-CR). A frameshift mutation was found in the *ND3* gene. Phylogenetic analysis illustrated monophyly of the subfamily Melieraxinae with high statistical support. The genetic resource obtained here will help to resolve taxonomic issues related to subspecies of *M. migrans* and will act as a starting point for conservation genetics.

The black kite, *Milvus migrans*, is a medium-sized bird of prey in the family Accipitridae and one of the most common diurnal raptors (Orta et al. 2017). It occurs throughout the Old World and Australasia (Sergio and Boto 1999). This species has experienced local declines in some regions, such as Portugal, Eastern Europe, and Russia, while remaining stable or increasing in Western Europe (Bustamante and Hiraldo 1990; Vinuela and Sunyer 1994; Sergio and Boto 1999). It is a winter migratory bird, although some individuals are considered year-round residents. The black kite is listed as a species of Least Concern (LC) on the International Union for the Conservation of Nature Red List of Threatened Species (BirdLife International 2016). However, in South Korea, this species has been classified as an endangered species II by the Ministry of Environment of Korea (NIBR 2011). In the present study, we sequenced and characterized the mitogenome of *M*. *migrans*.

An *M. migrans* sample (IN1594) was collected from Myongji-dong, Kangseo-gu, Busan, South Korea and deposited in the National Institute of Biological Resources at Inchoen, South Korea. Total genomic DNA was extracted from the muscle tissue using a DNeasy^®^ Blood & Tissue Kit (Qiagen, Hilden, Germany). The complete mitogenome was determined using next generation sequencing (18,016 bp in length; GenBank MG930481). Annotation was performed with the online tool DOGMA (Wyman et al. 2004) and the software ARWEN (Laslett and Canbäck 2008). The complete mitogenome of *M. migrans* contained 13 protein-coding genes (PCGs), 22 tRNAs, 2 rRNAs, a putative control region (CR), and a second control region (pseudo-CR). The pseudo-CR (869 bp) was located between tRNA-Glu and tRNA-Phe. The PCGs were located on the H-strand, with the exception of *ND* and eight tRNA genes that were transcribed from the L-strand. The overall base composition of the mitogenome was 30.0% A, 32.5% C, 17.3% G, and 23.8% T; thus, the percentage of A and T (53.8%) was slightly higher than that of G and C (46.2%). Most of the protein-coding genes started with an ATG start codon, except *COX I* and *ND5*, which had GTG as an initiation codon. As is frequently reported for Accipitridae species (9 species of the 15 examined in this study, Figure 1), a frameshift mutation (insertion of “C”) in the *ND3* gene was also found, which was located at position 174.

**Figure 1.**
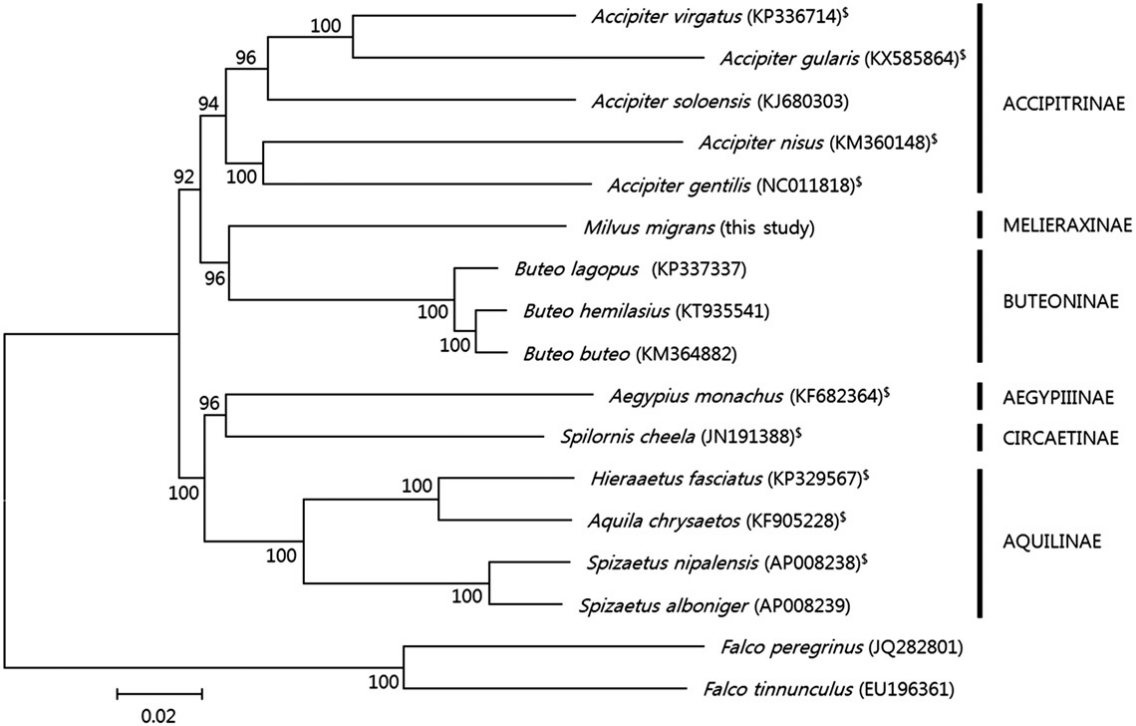
Phylogenetic tree of Acciptridae species based on the concatenated nucleotide sequences of the 13 protein-coding genes of their mitochondrial genomes. The analysis was performed using the MEGA 6.0 (Tamura et al. 2013) software, and bootstrap values are shown at the nodes. GenBank accession numbers for the sequences are indicated next to species designations. “$” indicates a species with a frameshift mutation in *ND3*.

A phylogenetic analysis (Figure 1) performed using the 13 PCGs of the *M. migrans* mitogenome and additional available Accipitridae sequences retrieved from GenBank demonstrated the monophyly of the subfamily Melieraxinae with high statistical support. The Melieraxinae clustered closely to the Buteoninae, which is inconsistent with the results of Lerner and Mindell (2005), which showed that Accipitrinae is closer to Melieraxinae. The complete mitogenome of *M. migrans* provides genetic resources that can be used to address existing taxonomic issues regarding the subspecies of *M. migrans* and that can be applied to conservation genetics.

## References

[CIT0001] BirdLife International 2016 *Milvus migrans* The IUCN Red List of Threatened Species 2016: e.T22734972A95097654. [accessed 2017 Nov 27]. 10.2305/IUCN.UK.2016-3.RLTS.T22734972A95097654.en.

[CIT0002] Bustamante J, Hiraldo F. 1990 Adoptions of fledglings by black and red kites. Animal Behav. 39:804–806.

[CIT0003] Laslett D, Canbäck B. 2008 ARWEN: a program to detect tRNA genes in metazoan mitochondrial nucleotide sequences. Bioinformatics. 24:172–175. 18033792 10.1093/bioinformatics/btm573

[CIT0004] Lerner HRL, Mindell DP. 2005 Phylogeny of eagles, old world vultures, and other accipitridae based on nuclear and mitochondrial DNA. Mol Phylogenet Evol. 37:327–346. 15925523 10.1016/j.ympev.2005.04.010

[CIT0005] National Institute of Biological Resources (NIBR) (2011). Red Data Book of Endangered Birds in Korea. Incheon, Korea: National Institute of Biological Resources (NIBR) (In Korean).

[CIT0006] Orta J, Marks JS, Garcia EFJ, Kirwan GM. 2017 Black Kite (*Milvus migrans*) In: del Hoyo J, Elliott A, Sargatal J, Christie DA, and de Juana E, editors. Handbook of the Birds of the World Alive. Barcelona: Lynx Edicions (Retrieved from https://www.hbw.com/node/52978 on 21 November 2017).

[CIT0007] Sergio F, Boto A. 1999 Nest dispersion, diet, and breeding success of Black Kites (*Milvus migrans*) in the Italian pre-Alps. J Raptor Res. 33:207–217.

[CIT0010] Tamura K, Stecher G, Peterson D, Filipski A, Kumar S 2013 MEGA6: Molechular Evolutionary Genetics Analysis Version 6.0. Mol Biol Evol. 30:2725–2729. 24132122 10.1093/molbev/mst197 PMC3840312

[CIT0008] Vinuela J, Sunyer C. 1994 Black kite, *Milvus migrans* In: Tucker GM and Heath ME, editors. Birds in Europe: their conservation status. BirdLife International (Conservation Series No. 3). Cambridge, UK: European Environment Agency p. 148–149.

[CIT0009] Wyman SK, Jansen RK, Boore JL. 2004 Automatic annotation of organellar genomes with DOGMA. Bioinformatics. 20:3252–3255. 15180927 10.1093/bioinformatics/bth352

